# Cross-cultural adaptation and psychometric properties of the child perceptions questionnaire 11-14 (CPQ11-14) for the peruvian spanish language

**DOI:** 10.4317/medoral.18975

**Published:** 2013-05-31

**Authors:** Jenny Abanto, Ursula Albites, Marcelo Bönecker, Saul Martins-Paiva, Jorge L. Castillo, Denisse Aguilar-Gálvez

**Affiliations:** 1 Pediatric Dentistry and Orthodontics Department, Dental School, University of São Paulo-USP, Brazil; 2 Pediatric Dentistry Department, Dental School, Científica del Sur University - UCSUR, Peru; 3 Chairman Professor of Pediatric Dentistry and Orthodontics Department, Dental School, University of São Paulo-USP, Brazil; 4 Pediatric Dentistry and Orthodontics, Dental School, Federal University of Minas Gerais -UFMG, Brazil; 5 Pediatric Dentistry Department, Dental School, Universidad Peruana Cayetano Heredia - UPCH, Peru; 6 Chairman Professor of Pediatric Dentistry Department, Dental School, Universidad Científica del Sur - UCSUR, Peru

## Abstract

Objectives: Oral-Health-Related Quality of Life (OHRQoL) instruments, such as the Child Perceptions Questionnaire 11-14 (CPQ11-14), are broadly used in oral health surveys around the world. However, there is a lack of these instruments in Spanish language limiting the comparison of OHRQoL outcomes among countries, cultures and ethnic groups. The aim of the present study was to cross-culturally adapt the CPQ11-14 to the Peruvian Spanish language and assess its reliability and validity. Material and Methods: To test the translation and cross-cultural adaptation, 60 children aged 11-to-14-years answered the CPQ11-14 in two pilot tests. After that, the questionnaire was tested on 200 children of the same age, who were clinically examined for dental caries. The internal consistency was assessed by Cronbach’s alpha coefficient while repeat administration of the CPQ11-14 on the same 200 children facilitated the test-retest reliability via intraclass correlation coefficient (ICC). Construct and discriminant validity were based on associations of the CPQ11-14 with global ratings of oral health and clinical groups respectively. Results: The mean (standard deviation) CPQ11-14 score was 20.18(13.07). Internal consistency was confirmed by a Cronbach’s alpha of 0.81. Test-retest reliability revealed excellent reproducibility (ICC= 0.92). Construct validity was confirmed demonstrating statistically significant associations between total CPQ11-14 score and global ratings of oral health (p=0.035) and overall well-being (p<0.001). The measure was also able to discriminate between children with dental caries experience and those without (mean scores: 26.32 and 12.96 respectively; p<0.001). Conclusions: The Spanish CPQ11-14 has satisfactory psychometric properties and is applicable to children in Peru.

** Key words:**Oral health, quality of life, children, adolescent, validity, reliability.

## Introduction

The concept of oral health-related quality of life (OHRQoL) relates to the impact that oral conditions have on the individual’s daily functioning, well-being or quality of life. During the last ten years, researchers have developed different self-reported OHRQoL instruments for children and adolescents among 8 and 15 years old (1-4). Of these instruments, the most employed, as supplement to clinical indicators to assess the child’s OHRQoL, is the Child Perceptions Questionnaire 11-14 (CPQ11-14) (1). Its validity has been demonstrated in English-speaking children in Canada, United Kingdom and New Zealand, in Danish in Denmark, German in Germany, Arabic in Saudi Arabia, Portuguese in Brazil, Thai and Chinese languages (1,5-12).

The lack of the CPQ11-14 in Spanish language limits the use of this standard measure for assessing OHRQoL outcomes in different cultural and ethnic groups and, precludes comparisons with data from other parts of the world. In addition, OHRQoL instruments have a potential role as outcome measures for evaluating service initiatives and oral health promotion programs, the perceptions of the impact of dental disorders on the quality of life are likely to trigger demand for dental treatment. In that respect, the use of the CPQ11-14 on children is essential, particularly in developing countries like Peru, where the prevalence of untreated dental caries at 12-year-old is high (83,3%) (13).

Therefore, the aim of the present study was to carry out the cross-cultural adaptation of the CPQ11-14 to the Peruvian Spanish language and to test its reliability and validity in Peruvian 11 to 14 years old children.

## Material and Methods

-Description of the Child Perceptions Questionnaire 11-14 (CPQ11-14) 

The CPQ11–14 is a specific questionnaire for assessing the impact of oral health conditions on the quality of life of 11 to 14-year-old children (14). The items address the frequency of events in the previous three months. It is structurally composed of 37 items distributed among 4 domains: oral symptoms (6 items), functional limitation (10 items), emotional well-being (9 items) and social well-being (12 items). A 5-point Likert scale is used, with the following options: ‘Never’ = 0; ‘Once/twice’ = 1; ‘Sometimes’ = 2; ‘Often’ = 3; and ‘Every day/almost every day’ = 4.

The CPQ11–14 scores are calculated as a simple sum of the response codes. Scores for each of the four domains can also be computed. Since there were 37 questions, the final score can vary from 0 to 148, for which a higher score denotes a greater degree of the impact of oral conditions on the quality of life of the child.

The authors also designed two questions asking the children for a global rating of their oral health and the extent to which their oral health affected their overall well-being was obtained (14). These questions are: ‘Would you say that the health of your teeth, lips, jaws and mouth is...?’ and ‘How much does the condition of your teeth, lips, jaws or mouth affect your life overall?’ These global ratings had a five-point response format. The responses were scored as follows: for global rating of oral health, (0) excellent, (1) very good, (2) good, (3) fair and (4) poor; and for overall well-being, (0) not at all, (1) very little, (2) somewhat, (3) a lot and (4) very much.

-Translation and Adaptation of the CPQ11–14 

The CPQ11–14 items were translated and adapted to Spanish for Peru according to published guidelines (15-17). Based on these standard guidelines, two initial translations were made independently by two translators; they are Peruvians fluent in the English language, who had lived more than four years in English speaking countries, did not know the objectives of the study and had experience in OHRQoL studies. Both translations were reviewed in a consensus meeting in Peru. The Revision Panel for this meeting consisted of four postgraduate professors, all fluent in both Spanish and English, who knew the objectives of the study and had experience in OHRQoL studies (17). For the determination of conceptual and item equivalence, the Revision Panel evaluated this version and compared it to the original. Attention was given to the meaning of the words in the different languages in order to retain content similarity in the different cultures. An effort was made to identify possible difficulties in understanding the questionnaire. A consensus forward-translated version was developed as a result of this process.

This draft synthesis forward-translated version of the CPQ11–14 was then pilot-tested on a convenience sample of forty 11–14 years-old children. Modifications were made according to children’s comments, in order to clarify the content of the questionnaire. Children individually suggested the substitution of some words and expressions by their synonyms in order to facilitate comprehension. The Revision Panel developed a first Peruvian version as a result of this process.

In order to check the translation, this first version was then independently translated back into English by two native English-speaking translators fluent in the Spanish language, who lived more than four years in Peru, were not previously involved in the study and with experience in health questionnaire translations. These two back-translated English versions proved nearly identical. The Revision Panel compiled a single version of these translations as a result of this process. To determine semantic equivalence, a group composed of three dental surgeons fluent in both languages and with no prior knowledge of the study compared the back-translated English version with the original English version. The aim of this step was to achieve a “similar effect” on respondents who speak two languages (English and Spanish).

This draft of the first version of the CPQ11–14 was then pilot-tested for a second time on a convenience sample of twenty 11–14 years-old children (different from the ones involved in the first pilot), five for each age. There were no changes in terms of new suggestions or difficulties of comprehension. This second pilot testing verified the appropriateness and cultural relevance of the target language version. Finally, the Revision Panel formulated the final Peruvian version as a result of this process.

**Figure 1:**
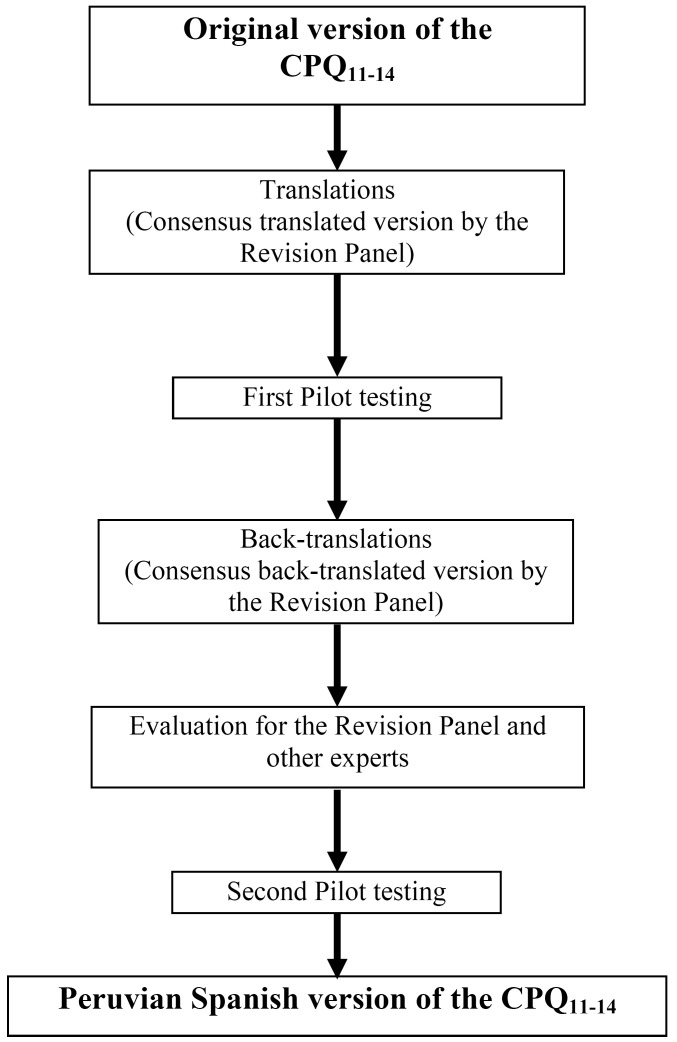
Flow chart of the cross-cultural adaptation and translation steps.

Mode of administration of the Peruvian version of the CPQ11–14 was made in face-to-face independent interviews in order to reduce losses stemming from self-application and also to avoid the influence of parents in their children’s responses. Structures, instructions, mode of administration and measurement methods of the questionnaire were similar to the original English version of the CPQ11–14. The steps of the adaptation and translation process are presented in a flow chart (Fig. 1).

-Assessment of validity and reliability

The Peruvian version of the CPQ11-14 (See Additional file 1) was administered in face-to-face independent interviews to 200 chil-dren between the ages of 11 and 14 years in four schools, two of them were public schools in a deprived area and the others private schools in a privileged area. All schools were located in the city of Lima, central part of Peru. Children were randomly selected from official school registries. Children that had not received dental treatment during the study and had no systemic diseases were eligible for inclusion in the study. In order to assess test-retest reliability, the 200 children completed the CPQ11-14 for a second time, 7 to 14 days after the first interview. The study was approved by the Human Research Ethics Committee of the University of São Paulo.

Interviews were carried out on the same day of dental screening before the clinical oral examinations by three-trained interviewers who were blind to the oral screening examinations findings. The interviewers were trained in the administration and intonation of each question of the Peruvian CPQ11-14. They were also clearly instructed to avoid suggesting responses and to show the answering options while reading them. The children’s oral examinations referred to dental caries assessment according to Knutson criteria (18). To assess discriminant validity, children were divided into two clinical groups: those with no dental caries experience (DMFT=0) vs. those with dental caries experience in one or more teeth (DMFT ?1) (18). The children’s oral examinations were carried out by a single specialist in pediatric dentistry who was previously trained and calibrated (Kappa intra-agreement= 0.92).

-Data analysis

The SPSS software program (version 17.0 SPSS Inc., Chicago, IL, USA) was used for data analysis. Initially descriptive analyses were performed to assess the prevalence of oral impacts and measures of central tendency (means and standard deviations) of total and individual domain scores of the Peruvian CPQ11-14.

Internal consistency of the CPQ11-14 was assessed using Cronbach’s alpha, inter-item and item-total correlation coefficients. Test-retest reliability was assessed by calculating the Intraclass Correlation Coefficient (ICC) with a two-way random effects model for the CPQ11-14 score using the data from the same 200 children who were interviewed a second time by the same interviewer.

To test the construct validity, correlations between the scores of each domain, total score and global ratings were analyzed using Spearman’s correlation coefficient. Discriminant validity was tested by comparing the mean CPQ11-14 scores between children with carried experience and those without (DMFT=0 vs. DMFT ?1). As the scores CPQ11-14 scores were not normally distributed, the nonparametric Mann-Whitney test was used to evaluate the difference in mean scores between the two groups. The level of significance was set at 0.05.

## Results

A total of 243 children were invited to participate in the validation study. Of them, 43 were not included because they did not conform to the study criteria and of the 200 that were eligible, 200 provided signed parental informed consent, resulting in a response rate of 82.3%.

The sample (n=200) consisted of 108 (54.0%) girls and 92 (46.0%), of whom 95 (47.5%) were from public schools and 105 (52.5%) from private schools. The mean (standard deviation) age of children was 12.5 (1.12). A total of 92 children (46.0%) had no caries experience (DMFT= 0) and 108 (54.0%) had experience of dental caries (DMFT? 1).

All questionnaires were fully completed. The scores for the total scale in the study population ranged from 0 to 69, with a mean (standard deviation) of 20.18 (13.07). All children (100%) in the sample reported oral impacts when “never threshold (CPQ11-14 score> 0)” was considered, 99.5% and 99.0% when “sometimes threshold (CPQ11-14 score> 2)” and “often threshold (CPQ11-14 score> 3)” were considered. Considering the “never threshold”, 194 (97.0%) reported experiencing oral symptoms in the previous 3 months; 197 (98.5%) reported functional limitations; 184 (92%) reported emotional well-being impacts and 174 (87%) reported social well-being impacts.

-Reliability 

The Cronbach’s alpha coefficient was 0.81 for the total score and ranged from 0.50 for oral symptoms to 0.75 for social well-being domains ( Table 1). Test-retest reliability was assessed using the ICC, which was 0.92 for the total score ranging from 0.90 for emotional well-being to 0.93 for social well-being domains ( Table 1).

**Table 1:**
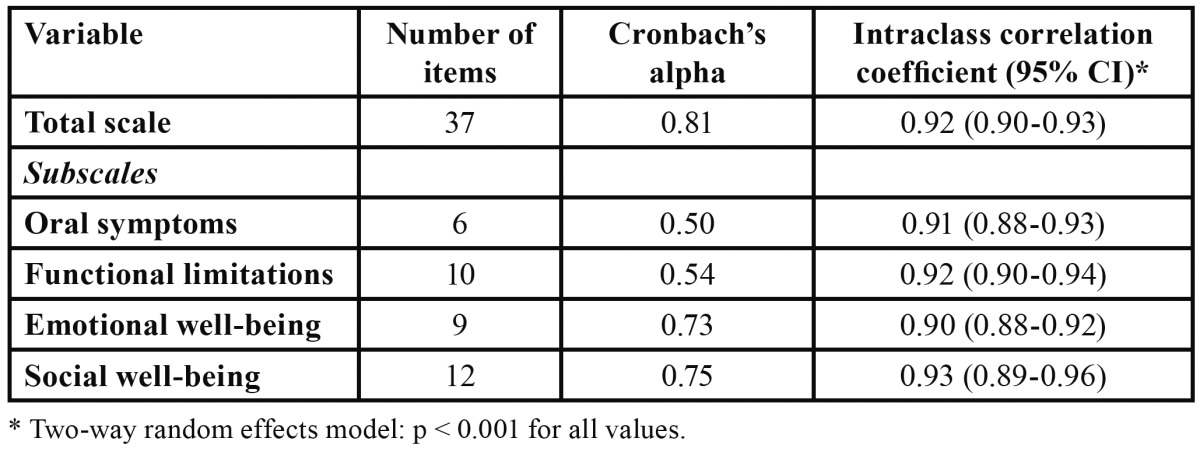
Reliability statistics for total score and subscales (n = 200).

-Construct validity

The correlations between the global ratings (oral health and overall well-being) and the total scale (r= 0.195 and r= 0.306), functional limitations domain (r= 0.190 and r= 0.186), emotional well-being domain (r= 0.154 and 0.313 and social well-being domain (r= 0.145 and 0.249), were mediocre but statistically highly significant ( Table 2). Oral symptoms domain was only significant associated with the global rating of overall well-being (r= 0.260), but not oral health (r= 0.198) ( Table 2).

**Table 2:**
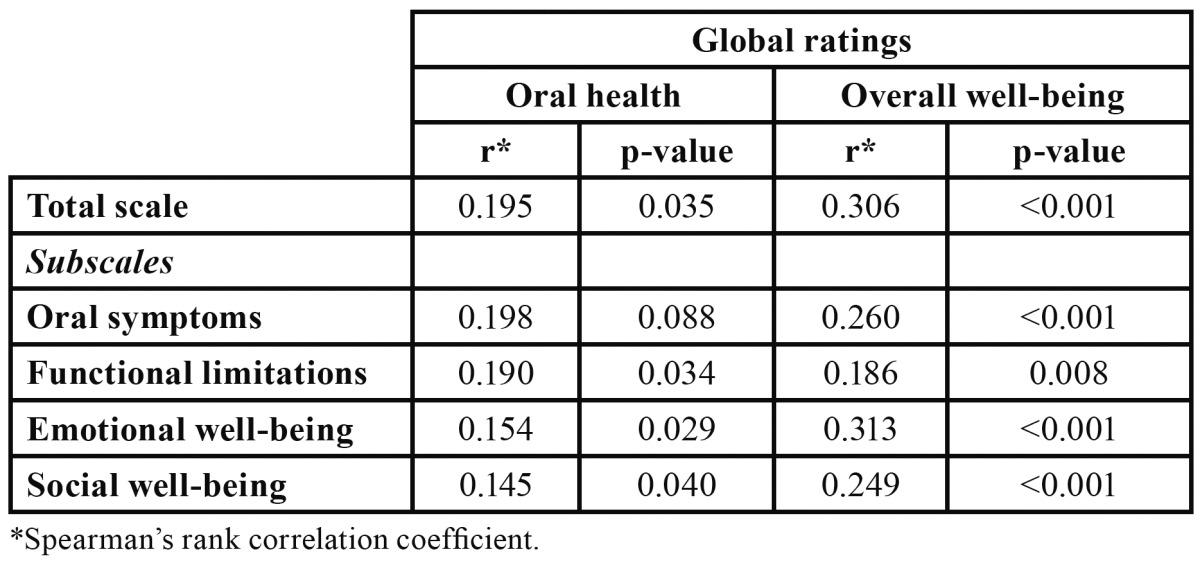
Construct validity: rank correlations between total scale and subscale scores, and global rating of oral health and overall well-being (n = 200).

-Discriminant validity

There was a significant difference in total and domain scores of the CPQ11-14 between children without dental caries experience and those with dental caries experience in one or more teeth ( Table 3).

**Table 3:**
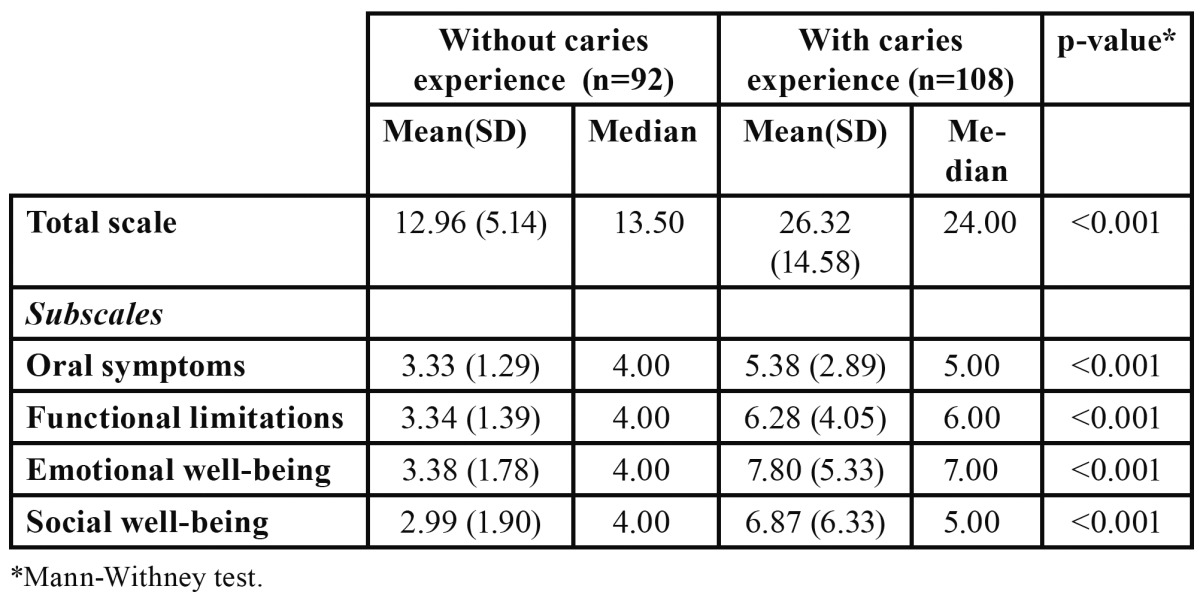
Discriminant validity: overall and subscales scores for children without dental caries experience and those with dental caries experience.

## Discussion

This research carried out the cross-cultural adaptation and validation of the CPQ11-14 to the Peruvian Spanish language. To the best of our knowledge, this is the first study that adapted and evaluated the psychometric properties of this measure in another language than the original English version for its further use in epidemiological surveys.

Whenever an OHRQoL measure is used in a different context or country population, the cross-cultural adaptation to the new language and its psychometric properties should be evaluated. Cross-cultural adaptation is a challenge that researchers must confront due to the influence of wider social context including family environment, friends, schools and cultural customs in different countries (19), however it is necessary because it allows that the validity and reliability of a measure be similar to the original. This study placed emphasis on the meticulous application of established guidelines for translation and cross-cultural adaptation of health-related quality of life measures (15-17). Applying careful inclusion criteria for the selection of the translators of the instrument and members of a Revision Panel in the target country ensures the accuracy of the translations therefore facilitating the conceptual equivalence with the original version of CPQ11-14. In addition to the meticulous translation, we employed a pretest phase that included cognitive interviews to show whether items were comprehensible and acceptable. Such pre-testing is critical for identifying potential problems with the questionnaire content, such as misunderstandings about the intended meaning of the items, their clarity and cultural relevance. As such, this phase has an important contribution to the cross-cultural adaptation of the target language version of a measure. The results achieved from this methodological design show that despite ethnic and cultural differences the CPQ11-14 achieved the same semantic equivalence in English and Peruvian Spanish language. Furthermore, we tested the measure in children from different background, public schools in a deprived area and private schools in a privileged area, and all children in our study were able to understand the Peruvian CPQ11-14 and respond appropriately to the questions.

The Peruvian Spanish version of the CPQ11-14 exhibited acceptable validity and reliability, thus indicating its use for child popula-tions of similar ages in Peru. Test-retest reliability was confirmed by the ICC, which showed excellent correlations between responses in the first and second time interviews for the total scale and domains. Cronbach’s alpha coefficient was 0.81 for the total scale, indicating adequate internal reliability, as reliability of 0.5 or above is considered acceptable (20). For the domains, the coefficient ranged from 0.50 for ‘oral symptoms’ to 0.75 for ‘social well-being’ domains. Similar results were found in the original version of the instrument (1).

It was also demonstrated the ability of the Peruvian CPQ11-14 to significantly discriminate between different clinical groups according to caries experience. Similar associations were found in other versions assessing the validation of the CPQ11-14; however, comparisons cannot be done due to the use of different indices for evaluating and analyzing caries condition (1,5-12). In spite of the instrument was able to discriminate clinical groups, particular attention must be given to the high scores of the group without caries experience (teeth-healthy sample). This may have occurred mainly by the presence of other factors involved in this sample, such as socioeconomic factors, which may affect the responses and impact of OHRQoL measures (21,22). However, the assessment of these associations and confounding factors correspond to studies assessing the impact of oral diseases and other factors on OHRQoL, and this was not the aim of this validation study. Concerning construct validity, our findings on the associations of the CPQ11-14 score with the global ratings on oral health and overall well-being proved the validity of the measure, except between the oral symptoms domain and global oral health rating. Other versions of the CPQ11-14 did not also correlate some domains scores with the global oral health rating (1,5,7,8,10-12). A possible explanation is that the 11–14 year-olds consider their teeth to be healthy if caries-free or treated, while the global rating on overall well-being explore broader emotional and social aspects which may dominate in the minds of the 11–14 year-olds. Furthermore, the DMFT mean (standard deviation) in our sample was 2.02 (2.43), which is considered as low caries prevalence according to the WHO (23), thus it was expected no correlation with the oral symptoms domain.

Despite this is a convenience sample we tested the Peruvian version of the CPQ11-14 in different balanced socioeconomic groups represented by private and public schools from privileged and deprived areas, respectively. Moreover, the assessment of validity requires a sample size usually around 50 to 200 people in a cross-sectional design, while the assessment of test-retest reliability in a sub-sample (around 10% or 30 people) (15-17). Our sample was well within these arbitrary figures.

The cross-cultural adaptation and validation of the Peruvian Spanish version of the CPQ11-14 for pediatric patients has important implications for research and practice. Firstly, it can provide insight into the perceived impacts of oral diseases and disorders on the daily life of children and adolescents. Secondly, it could be used for clinicians to assess the success of oral treatment and could also potentially be a valuable outcome for evaluating public oral health programs and to weight the need of dental care services for this age group (24). The Spanish-language translation of the CPQ11–14 proved valid and reliable for its use on Peruvian children. However, to allow assessment of OHRQoL of a wider range of children, future studies aimed at validating the shorter and simpler version of the scale should be encouraged and administered in a population study.

## Conclusion

This study provides strong evidence supporting the cross-cultural validity of a Peruvian Spanish version of the CPQ11-14 that can be recommended as an OHRQoL measurement for Peruvian children aged 11-14 years.
